# Text summarization as a decision support aid

**DOI:** 10.1186/1472-6947-12-41

**Published:** 2012-05-23

**Authors:** T Elizabeth Workman, Marcelo Fiszman, John F Hurdle

**Affiliations:** 1 Department of Biomedical Informatics, University of Utah, HSEB 5775, Salt Lake City, UT 84112, USA; 2 National Library of Medicine, 8600 Rockville Pike, Building 38A, Room B1N-28 J, Bethesda, MD 20894, USA

## Abstract

**Background:**

PubMed data potentially can provide decision support information, but PubMed was not exclusively designed to be a point-of-care tool. Natural language processing applications that summarize PubMed citations hold promise for extracting decision support information. The objective of this study was to evaluate the efficiency of a text summarization application called Semantic MEDLINE, enhanced with a novel dynamic summarization method, in identifying decision support data.

**Methods:**

We downloaded PubMed citations addressing the prevention and drug treatment of four disease topics. We then processed the citations with Semantic MEDLINE, enhanced with the dynamic summarization method. We also processed the citations with a conventional summarization method, as well as with a baseline procedure. We evaluated the results using clinician-vetted reference standards built from recommendations in a commercial decision support product, DynaMed.

**Results:**

For the drug treatment data, Semantic MEDLINE enhanced with dynamic summarization achieved average recall and precision scores of 0.848 and 0.377, while conventional summarization produced 0.583 average recall and 0.712 average precision, and the baseline method yielded average recall and precision values of 0.252 and 0.277. For the prevention data, Semantic MEDLINE enhanced with dynamic summarization achieved average recall and precision scores of 0.655 and 0.329. The baseline technique resulted in recall and precision scores of 0.269 and 0.247. No conventional Semantic MEDLINE method accommodating summarization for prevention exists.

**Conclusion:**

Semantic MEDLINE with dynamic summarization outperformed conventional summarization in terms of recall, and outperformed the baseline method in both recall and precision. This new approach to text summarization demonstrates potential in identifying decision support data for multiple needs.

## Background

Clinicians often encounter information needs while caring for patients. Several researchers have studied this issue [1-6]. In their 2005 study, Ely and his colleagues discovered that physicians developed an average of 5.5 questions for each half-day observation, yet could not find answers to 41% of the questions for which they pursued answers [7]. Ely cited time constraints as one of the barriers preventing clinicians from finding answers. Chambliss and Conley also found that answer discovery is excessively time consuming; yet they also determined that MEDLINE data could answer or nearly answer 71% of clinicians’ questions in their separate study [8]. PubMed, the National Library of Medicine’s free source for MEDLINE data, was not exclusively designed to be a point-of-care information delivery tool. It generally returns excessive, often irrelevant data, even when implementing diverse search strategies [9]. Clinicians can spend an average of 30 minutes answering a question using raw MEDLINE data [10]. This is by and large due to the process of literature appraisal, which is naturally lengthened by excessive retrieval [11]. Thus this information discovery process is not practical for a busy clinical setting [10]. Applications that use natural language processing and automatic summarization of PubMed and present it in a compact form potentially can provide decision support data in a practical manner.

### Objective

The objective of this study was to evaluate the performance of a new automatic summarization algorithm called Combo in identifying decision support data. We hypothesized that a natural language processing application, enhanced with the algorithm, could identify intervention data which is also provided by a commercial decision support tool. To operationalize this pursuit, we incorporated the algorithm into Semantic MEDLINE [12], an advanced biomedical management application. We sought data on drug treatment and preventive interventions for four disease topics, and evaluated the results by comparing output to clinician-vetted reference standards based on recommendations from a commercial decision support product, DynaMed. The Combo system was also compared to a baseline as well as a conventional summarization method within the Semantic MEDLINE methodology.

### Related research

Natural language processing applications that summarize bibliographic text such as PubMed citations try to facilitate literature appraisal by providing succinct, relevant information suitable for point-of-care decision support. The objective of automatic text summarization is “to take an information source, extract content from it, and present the most important content to the user in a condensed form and in a manner sensitive to the user’s application’s need” [13]. Automatic text summarization can be applied to multiple documents or information sources [14], such as bibliographic citations retrieved from PubMed. Researchers have noted the potential value that summarized text holds in patient care. Previous research efforts provide interesting examples of approaches to summarizing PubMed and other text. Using a multimedia application called PERSIVAL, McKeown and her colleagues retrieved, ranked, and summarized clinical study articles (along with digital echocardiogram data) according to a patient’s profile information [15]. Article characteristics, specifically the properties of individual segments of text, were matched against information from a patient’s record. Within this process, the researchers used templates to identify and represent content. These templates identified six potential relations (risk, association, prediction, and their negations) existing between findings, parameters, and dependence properties. The results are then ranked according to potential relevancy to the specific patient’s information, consolidated, and presented to the user. To operate the clinical question answering application AskHERMES, Cao and his colleagues used a machine learning approach to classify questions, and they utilized query keywords in a clustering technique for presenting output [16]. AskHERMES draws answers from PubMed citations, in addition to eMedicine documents, clinical guidelines, fulltext articles, and Wikipedia entries. It uses a scoring system to assess similarity between text segments (adjacent sentence blocks) and the properties of clinical questions. Yang and his associates used a three-step pipeline to identify mouse gene information in PubMed data [17]. Using a topically-focused subset of PubMed, they tagged gene and protein names. They stored abstract and title sentences in a database, along with MeSH entries and other data. Each gene was modeled according to associated MeSH headings, Gene Ontology terms, and free text citation terms referencing the gene of interest. They clustered the data using these three features and a direct-k clustering algorithm. Sentences addressing specific genes were ranked, allowing a user to access the desired amount of sentences for review.

**Figure 1:**
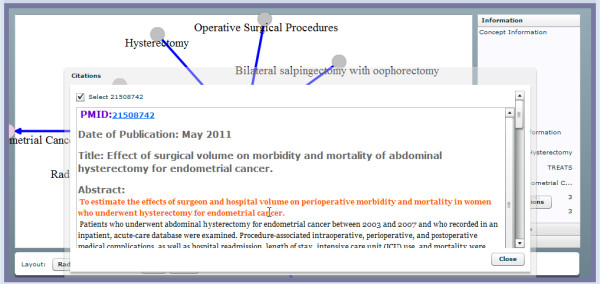
**Semantic MEDLINE visualization output.** The user has selected citations addressing hysterectomy as a treatment of endometrial cancer from the graph.

While these innovative summarization approaches have several strengths, their output lacks an explicit, deliberate point-of-view focus. A point-of-view is an additional concept such as treatment or genetic etiology. When summarized text is subjected to this additional conceptual refinement, system output may better address what type of information a clinician is seeking. This paper describes an application, Semantic MEDLINE with dynamic text summarization (i.e., enhanced with the Combo algorithm), which automatically identifies the prominent point-of-view reflected in the PubMed citations it receives as input, and refines output accordingly. Controlled vocabularies such as MeSH provide point-of-view filtering in basic information retrieval in the form of subheadings that can be incorporated into a search query. An integrated, semantic processor called SemRep identifies many argument-binding relations in text, assisting the summarization phase to accommodate several point-of-view refinements. Applications such as Semantic MEDLINE that utilize semantic predications have the advantage of presenting a compact expression of the original information that can be filtered according to a user’s specific information need, including desired point-of-view focus. Semantic predications are succinct subject_verb_object declarations that simplify the meaning of the PubMed text from which they are drawn [18]. Due to their structure, they are well suited for computational analysis [19]. To capture the rich and varied nature of bibliographic text, Semantic MEDLINE identifies many relations that bind subject and object arguments. Semantic MEDLINE is presented to users through a Web portal that combines information retrieval, semantic processing, automatic summarization, and visualization into a single application. A user activates Semantic MEDLINE by submitting a PubMed-style keyword or MeSH query. Semantic MEDLINE’s three individual components -- semantic processing (SemRep), summarization, and visualization -- transform MEDLINE text into concise declarations, filter these according to a user’s needs, and present the results in an informative graphic display (Figure 1).

### SemRep

SemRep [20] is a rule-based NLP application that interprets the meaning of abstract and title text in citations and transforms it into compact, subject_verb_object declarations known as semantic predications. It draws upon resources within the Unified Medical Language System (UMLS) [21] to accomplish this. For example, if the original text is:

"“These results suggest the possibility of molecular-targeted therapy using cetuximab for endometrial cancer” [22]"

SemRep produces:

(1) $$\text{cetuximab}\left|\text{phsu}\right|\text{TREATS}\left|\text{Endometrial carcinoma}\right|\text{neop}$$

In this example, SemRep identifies the subject and object of the original text as cetuximab and endometrial cancer, respectively. Using MetaMap [23] technology, it maps these terms to the corresponding UMLS Metathesaurus preferred concept terms cetuximab and Endometrial carcinoma, as indicated in the resulting semantic predication. Utilizing the UMLS Semantic Network, SemRep also identifies the most likely logical semantic types associated with the subject and object, which in this case are pharmacological substance (abbreviated as phsu) and neoplastic process (abbreviated as neop). SemRep also utilizes the UMLS Semantic Network to identify the relation, or predicate, that binds the subject and object. In this case, it is TREATS. SemRep identifies 26 such relations, plus their negations, in PubMed text. Additionally, SemRep identifies the four comparative predicates compared_with, higher_than, lower_than, and same_as [24].

### Summarization

Summarization in Semantic MEDLINE [25] filters SemRep output for a point-of-view concept and a seed topic concept selected by the user. The project described in this paper implemented a dynamic form of summarization. Here we describe both the dynamic and conventional summarization methods. Conventional Semantic MEDLINE offers summarization for five points-of-view: treatment of disease [26]; substance interaction [27]; diagnosis [28]; pharmacogenomics [29] and genetic etiology of disease [30]. For example, if the seed topic was Endometrial carcinoma and the point-of-view was treatment, summarization would identify semantic predications relevant to these paired concepts. Point-of-view concepts are similar to subheading refinements that can be combined with logical MeSH headings. For example, “Carcinoma, Endometrioid/therapy[MeSH]” could serve as a PubMed search query seeking citations addressing treatment options for endometrial carcinoma. Summarization accomplishes topic and point-of-view refinements of SemRep output by subjecting it to a four-tiered sequential filter:

Relevance: Gathers semantic predications containing the user-selected seed topic. For example, if the seed topic were Endometrial carcinoma, this filter would collect the semantic predication *cetuximab-TREATS-Endometrial carcinoma*, among others.

Connectivity: Augments Relevance predications with those which share a non-seed argument’s semantic type. For example, in the above predication *cetuximab-TREATS-Endometrial carcinoma*, this filter would augment the Relevance predications with others containing the semantic type “pharmacological substance” because it is the semantic type of the non-seed argument cetuximab.

Novelty: Eliminates vague predications, such as *pharmaceutical preparation-TREATS-patients*, that present information that users already likely know, and are of limited use. Such predications that Novelty filtering removes usually contain very general arguments that are of little use.

Saliency: Limits final output to predications that occur with adequate frequency. For example, if *cetuximab-TREATS-Endometrial carcinoma* occurred enough times, all occurrences would be included in the final output.

Operationalizing the points-of-view coverage of the summarization process can be done in one of two ways. Conventional summarization [29] requires creating separate applications known as schemas for each new point-of-view emphasis. This requires hard-coding specific subject_predicate_object patterns into the application, which limits output to predications matching the specific patterns for the new point-of-view. Prior to coding, designers must determine which patterns best capture semantic predications relevant to the given point-of-view. Conventional schema output may also be refined using degree centrality measurements [31]. The novel approach to summarization that we explore here is to produce saliency measurements on the fly, using a dynamic statistical algorithm known as Combo [19]. Combo adapts to the properties of each individual SemRep dataset by weighing term frequencies with three combined metrics. This flexibility enables summarization for multiple points-of-view, eliminates the work of hard-coding schemas, and uses a single software application.

### The Combo algorithm to support summarization

The Combo algorithm combines three individual metrics to identify salient semantic predications:

#### ***Kullback–Leibler Divergence***

The Kullback–Leibler Divergence (KLD) [32], as applied here, assesses the values of predicates in SemRep output originating from a search query that expresses a subject paired with a point of view, (distribution P) to SemRep data with only the subject focus (distribution Q):

(2) $$\text{D}\left(\text{P}||\text{Q}\right)=\sum \text{ P}\left(\text{x}\right){\text{log}}_{2}\left(\text{P}\left(\text{x}\right)/\text{Q}\left(\text{x}\right)\right)$$

Both distributions P and Q consist of relative frequencies for their respective predicates. Each predicate shared by each distribution receives a KLD value (before summing) indicating its value in conveying the point-of-view expressed in distribution P’s search query. A database of PubMed citations from the last 10 years processed with SemRep provides the distribution Q data. Prior to our research, the KLD metric performed well in a similar task involving predicate assessment [33].

#### *RlogF*

Riloff developed the RlogF metric [34] to assess the relevance of extracted patterns consisting of a syntactic constituent (i.e., a noun or verb phrase) and its arguments (i.e., a direct or indirect object):

(3) $$\text{RlogF}({\text{pattern}}_{\text{i}})={\text{log}}_{2}({\text{semantic type frequency}}_{\text{i}})*\text{P}(\text{relevant }|{\text{ pattern}}_{\text{i}})$$

We adapted RlogF to assess the value of a semantic type as paired with a predicate. The log of a semantic type’s absolute frequency (semantic type frequency_i_) is applied to the quotient of dividing that same frequency with the absolute frequency of all semantic types that are also paired with the predicate (pattern_i_). We use RlogF to appraise combinations of predicates and non-seed topic semantic types. Using the example above, in *cetuximab-TREATS-Endometrial carcinoma*, the seed topic “Endometrial carcinoma” has the semantic type “neoplastic process”. The opposing argument “cetuximab” has the semantic type “pharmacologic substance”. RlogF would assess the significance of “pharmacologic substance” as bound to the predicate TREATS. The RlogF metric has been noted for its efficiency in identifying important predicate and argument patterns [35].

#### *PredScal*

Because the KLD metric assesses all predicates, KLD scores express a relative value that spans a dataset of SemRep output. RlogF scores only appraise a semantic type associated with a single predicate. Raw RlogF scores often exceed KLD scores, so we created a new metric called PredScal to scale and smooth RlogF scores according to the spatial proportions of predicates in a given SemRep dataset:

(4) $$1/{\text{log}}_{2}\left(\text{c}\right)$$

Here, c represents the count of unique predicates. In rare cases where there is only one unique predicate, PredScal defaults to a value of 1.

We combine the three metrics to yield a product, which is the final Combo score:

(5) KLD*RlogF*PredScal

Combo summarization output consists of the four highest scoring semantic type_a__verb_semantic type_b_ Relevancy patterns (based on novel predications containing the summarization seed topic) and the four highest scoring Connectivity patterns (patterns sharing a non-seed topic argument’s semantic type from one of the high scoring Relevancy patterns).

In the Saliency phase, conventional summarization uses metrics developed by Hahn and Reimer [36] which appraise “weights” that are dependent on the predefined subject_verb_object patterns.

In contrast, dynamic summarization does not utilize such predetermined patterns; instead it applies the Combo algorithm to all novel predications in order to determine which are more prominent in the data.

### DynaMed

DynaMed is a decision support tool that provides intervention recommendations. In a recent study, it tied with two other products for highest ranked evidence-based decision support tool [37]. It draws upon the professional literature using a “Systematic literature surveillance” method in evaluating published results, using a tiered-ranking of study design types [38]. For example, here is an excerpt of the DynaMed pneumococcal pneumonia drug treatment recommendation text that we used [39]:

Medications:

· treat for 10 days

· penicillin

○ aqueous penicillin G 600,000 units IV every 6 hours (2 million units every 4-6 hours if life-threatening)

○ procainepenicillin G 600,000 units intramuscularly every 8–12 hours

○ penicillin V 250–500 mg orally every 6 hours

## Methods

### Disease topics

In consultation with a clinician, we selected the four following disease topics for data acquisition:

· Arterial hypertension

· Diabetes mellitus type 2

· Congestive heart failure

· Pneumococcal pneumonia

Each disease is a significant global health concern, and of interest to clinicians in many areas of the world. Collectively, they have an interesting variety of preventive interventions and treatment options.

### Data acquisition

We executed a single PubMed search query for each disease topic and point-of-view pairing, (i.e., drug treatment or prevention), using specific MeSH term and subheading combinations. The following lists indicate the exact MeSH terms and subheadings we used in forming these pairings:

MeSH Terms:

· Hypertension

· Diabetes Mellitus, Type 2

· Heart Failure

· Pneumonia, Pneumococcal

Subheadings:

· drug therapy

· prevention and control

For example, to acquire citations addressing drug treatment options for pneumococcal pneumonia, we executed the search phrase “Pneumonia, Pneumococcal/drug therapy[Mesh]”. To provide an evidence-based focus, we first restricted output to the publication types “clinical trials,” “randomized controlled trials,” “practice guidelines,” and “meta-analyses.” We then acquired citations for systematic reviews, using the publication type “review” and the keyword phrase “systematic review.” Realistically, a clinician could engage Semantic MEDLINE using anything from a general keyword search to a very sophisticated search utilizing many of PubMed’s search options. In addition to providing the initial topic/point-of-view pairing, this method of forming search queries also provided a middle ground within the spectrum of queries a clinician might actually use. We also restricted publication dates to coincide with the most recently published source materials DynaMed used in building their recommendations, which served as the base for our evaluative reference standards (described in detail below). We restricted the retrieval publication dates in order to not retrieve materials that DynaMed curators could not have reviewed in creating their own recommendations. These cutoff dates are indicated in the Results section tabular data. The eight total search queries resulted in eight separate citation datasets, each representing a pairing of one of the four disease topics with one of the two subheading concepts. We executed the eight search queries and downloaded all citations in the period of July - August 2011.

### Data processing

We processed each of the eight citation datasets separately with SemRep, then with Semantic MEDLINE utilizing the Combo algorithm. We also processed the four SemRep output datasets originating from the search queries that included the drug therapy subheading with conventional Semantic MEDLINE utilizing the built-in treatment point-of-view schema (i.e., with predetermined, hard-coded patterns). We used the following UMLS Metathesaurus preferred concepts as seed topics (required by Semantic MEDLINE) to summarize SemRep data originating from both disease/drug treatment and disease/prevention and control search query pairings:

· Hypertensive disease

· Diabetes Mellitus, Non-Insulin-Dependent

· Congestive heart failure (OR Heart failure)

· Pneumonia, Pneumococcal

### Reference standard

We built a reference standard for each disease topic/point-of-view pairing, using vetted interventions from DynaMed, a commercial decision support product. We captured the DynaMed text for recommendations on both preventive and drug treatment interventions for each disease topic. We forwarded this text to two physician-reviewers, who highlighted the interventions they thought were viable for the associated diseases. In annotating these materials, we instructed the reviewers to ask themselves “What are the drugs used to treat this disease?” and “What interventions prevent this disease?”. Disagreements between the two annotators were forwarded to a third physician adjudicator, who made the final decision regarding the conflicting annotations. The two primary reviewers were a cardiologist and a preventive medicine specialist. The adjudicator was a pathologist. We measured agreement between the two reviewers using fundamental inter-annotator agreement (IAA) where instances of agreement are divided by the sum of agreement instances and disagreement instances, or in other words, matches/(matches + non-matches). As an example, we list below the final reference standard of DynaMed arterial hypertension preventive interventions:

· Maintain normal body weight

· Reduce sodium intake

· Increased daily life activity

· Higher folate intake

· Regular aerobic physical activity

· Diet reduced in saturated and total fat

· Walking to work

· Increased plant food intake

· Diet rich in fruits, vegetables and low- fat dairy products

· Relaxation

· Whole-grain intake

· Regular tea consumption

· Limit alcohol use

The final, combined reference standards included a total of 225 interventions, with an average of approximately 28 interventions for each disease topic/point-of-view pairing. Table 1 lists the counts for all eight reference standards.

**Table 1:** Reference standard intervention counts

	**Drug Treatment**	**Prevention**
Arterial Hypertension	27	14
Diabetes Mellitus Type 2	55	20
Congestive Heart Failure	59	16
Pneumococcal Pneumonia	31	3

### Baselines

We built eight baselines that simulated what a busy clinician might find when directly reviewing the PubMed citations. This is based on techniques developed by Fiszman [26] and Zhang [31]. To build baselines for the four disease topic/drug treatment pairings, we processed their PubMed citations with MetaMap, restricting output to UMLS Metathesaurus preferred concepts associated with the UMLS semantic group Chemicals and Drugs, and removed vague concepts using Novelty processing. Threshold values were determined by calculating the average mean of term frequencies in a baseline group, and then adding one standard deviation to the mean. In each group, all terms whose frequency scores exceeded the threshold value were retained to form the group’s baseline. For example, for the congestive heart failure drug treatment group, the method extracted 1784 terms that occurred 63924 times in the MetaMap data, with a mean of approximately 35.8 occurrences per term, and a standard deviation of 154.4. This produced a cutoff threshold of 190.3. Therefore, all MetaMap terms that occurred 190 times or more were included in the congestive heart failure drug treatment baseline (a total of 72 terms). This method is meant to simulate the types of terms a busy clinician might notice when quickly scanning PubMed citations originating from a search seeking drug treatment for a given disease.

We formed baselines for citations emerging from each disease topic/prevention and control pairing in a similar manner. We extracted the lines from the associated PubMed citations that contained the phrases “prevent,” “prevents,” “for prevention of,” and “for the prevention of.” These lines were processed with MetaMap, and all UMLS Metathesaurus preferred concepts associated with the UMLS disorders semantic group were removed, since the focus was preventive interventions and not the diseases themselves. Threshold values were calculated for the remaining terms, and those whose frequencies exceeded their threshold scores were retained as baseline terms. To reiterate, preventive baselines (as well as the drug treatment baselines) are meant to simulate what a busy clinician might notice when seeking interventions while visually scanning PubMed citations originating from a search seeking such interventions for a given disease.

### Comparing outputs to the reference standards

We evaluated outputs for the two summarization methods (Combo algorithm and conventional schema summarization) and the baselines by manually comparing them to the reference standards for the eight disease topic/subheading pairings. Since the reference standard was always a list of interventions, the comparison was straightforward. We measured recall, precision, and F_1_-score (balanced equally between recall and precision).

For both summarization systems, we measured precision by grouping subject arguments by name and determining what percentage of these subject groups expressed a true positive finding. For outputs for the four disease topic/drug intervention pairings, we limited analysis to semantic predications in the general form of “Intervention X_TREATS_disease Y”, where the object argument reflected the associated disease concept. If the subject intervention X argument matched a reference standard intervention, that intervention received a true positive status. In similar predications where the subject argument was a general term, such as “intervention regimes,” we examined the original section of citation text associated with the semantic predication. If this citation text indicated a reference standard intervention it received a true positive status. For example, in the dynamic summarization output for arterial hypertension prevention, the semantic predication “Dietary Modification_PREVENTS_Hypertensive disease” summarized citation text that included advice for dietary sodium reduction [40]; therefore, the reference standard intervention “reduce sodium intake” received a true positive status.

Only the Combo algorithm summarized output for the four disease topic/prevention and control pairings was compared to the reference standard, since there is no conventional schema for prevention. In addition to predications in the form “Intervention X_PREVENTS_disease_Y,” other predications where argument concepts had prevention terms such as “Exercise, aerobic_AFFECTS_blood pressure” and “Primary Prevention_USES_Metformin” were used, because their value was confirmed in a previous study [41].

We evaluated each baseline by comparing its terms to those of its associated reference standard. If a term in a baseline matched an intervention in the relevant reference standard, the baseline term received a true positive status. We also assigned true positive status to less specific baseline terms if they could logically be associated with related reference standard interventions. For example, in the baseline for pneumococcal pneumonia prevention the term “Polyvalent pneumococcal vaccine” was counted as a true positive, even though it did not identify a specific polyvalent pneumococcal vaccine that was in the reference standard.

## Results

The PubMed search queries retrieved varying quantities of output, as did SemRep, conventional, and dynamic summarization. Table 2 lists PubMed output citation quantities as well as retrieval cutoff dates according to disease topic and point-of-view. Citation quantities significantly vary; the arterial hypertension drug treatment dataset of 12335 included the most citations, whereas the pneumococcal pneumonia prevention dataset contained only 81 citations, less than one percent of the hypertension drug treatment citations. Overall, the search queries addressing prevention garnered far fewer citations than those seeking drug treatment data. In terms of retrieval by disease, search queries addressing pneumococcal pneumonia retrieved the least amount of citations.

**Table 2:** Citation retrieval results, with cutoff retrieval dates in parentheses

	**Drug Treatment**	**Prevention**
Arterial Hypertension	12335 (2010/08/31)	875 (2010/08/31)
Diabetes Mellitus Type 2	3716 (2010/06/30)	435 (2010/01/31)
Congestive Heart Failure	3256 (2010/12/31)	344 (2010/12/31)
Pneumococcal Pneumonia	115 (2008/12/31)	81 (2010/11/30)

Table 3, Table 4, and Table 5 list quantitative outputs for SemRep, Combo-enhanced dynamic summarization, and summarization using the conventional treatment schema. SemRep outputs reflect the size of the citation datasets received as inputs, with the arterial hypertension drug treatment dataset resulting in the most semantic predications (94353) and the pneumococcal pneumonia prevention dataset resulting in the least (643). The outputs for Combo and conventional summarization also reflect this trend. The conventional schema output was less than that of dynamic summarization for drug treatment data, for all four disease topics.

**Table 3:** SemRep semantic predication outputs

	**Drug Treatment**	**Prevention**
Arterial Hypertension	94353	4836
Diabetes Mellitus Type 2	37962	2654
Congestive Heart Failure	28951	2630
Pneumococcal Pneumonia	918	643

**Table 4:** Combo algorithm-enhanced summarization semantic predication output

	**Drug Treatment**	**Prevention**
Arterial Hypertension	13015	279
Diabetes Mellitus Type 2	3237	188
Congestive Heart Failure	4175	207
Pneumococcal Pneumonia	189	137

**Table 5:** Conventional treatment schema semantic predications output

	**Drug Treatment**
Arterial Hypertension	8052
Diabetes Mellitus Type 2	2645
Congestive Heart Failure	2375
Pneumococcal Pneumonia	62

### System performance

Performance metric outcomes are listed in Tables 6 and Table 7. Dynamic summarization performance exceeded conventional summarization for all drug treatment disease topics in recall; however, conventional summarization achieved better precision. No conventional schema is available in summarizing for a prevention point-of-view; therefore, just the Combo algorithm enhanced summarization and the baseline method performance outcomes are included in Table 7. Both dynamic and conventional summarization regularly outperformed the baseline method. These findings are discussed in the following section, including an error analysis addressing false positives and false negatives, suggesting adjustments that would significantly increase precision.

**Table 6:** Performance Metrics, Drug Treatment Point-of-View, for Combo-enhanced dynamic summarization (DS), conventional treatment schema (TS), and baseline (BL) methodologies

**Disease**	**Recall**			**Precision**			**F_1_-Score**		
	**DS**	**TS**	**BL**	**DS**	**TS**	**BL**	**DS**	**TS**	**BL**
Arterial	0.93	0.82	0.26	0.39	0.73	0.41	0.55	0.77	0.32
Hypertension									
Diabetes	0.89	0.56	0.35	0.35	0.68	0.25	0.50	0.62	0.29
Mellitus Type 2									
Congestive	0.93	0.70	0.13	0.34	0.60	0.25	0.50	0.64	0.17
Heart Failure									
Pneumococcal	0.65	0.26	0.19	0.43	0.83	0.32	0.51	0.39	0.24
Pneumonia									

**Table 7:** Performance Metrics, Prevention Point-of-View, for Combo-enhanced dynamic summarization (DS), and baseline (BL) methodologies

**Disease**	**Recall**		**Precision**		**F_1_-Score**	
	**DS**	**BL**	**DS**	**BL**	**DS**	**BL**
Arterial	0.77	0.23	0.13	0.13	0.22	0.17
Hypertension						
Diabetes	0.68	0.18	0.50	0.33	0.58	0.24
Mellitus Type 2						
Congestive	0.50	0.33	0.30	0.31	0.37	0.32
Heart Failure						
Pneumococcal	0.67	0.33	0.39	0.22	0.49	0.26
Pneumonia						

### Inter-annotator agreement

The annotations of the two reviewers resulted in an average IAA score of 0.54. Agreement was higher for all disease topics in terms of Drug Treatment ratings than Prevention ratings, with the exception of pneumococcal pneumonia. This generally parallels system recall performance, which is discussed further in the Discussion section. Table 8 lists all inter-annotator agreement scores.

**Table 8:** Inter-Annotator Agreement (IAA)

	**Drug Treatment**	**Prevention**
Arterial Hypertension	0.47	0.33
Diabetes Mellitus Type 2	0.73	0.44
Congestive Heart Failure	0.76	0.40
Pneumococcal Pneumonia	0.50	0.66

## Discussion

The results imply that dynamic text summarization with the Combo algorithm provides a viable alternative to direct review of PubMed citations for locating decision support data. This is encouraging, because dynamic summarization could expand the value of Semantic MEDLINE at the point-of-care. Performance improvements over the baseline methodology can be seen in both recall and precision results. Including findings from both drug treatment and prevention analyses, Combo produced average recall and precision scores of 0.75 and 0.35, while the baseline method yielded average recall and precision values of 0.25 and 0.28. Combo summarization outperformed the baseline methodology by an average F_1_-score margin of 0.21. The Combo algorithm especially performed well in terms of recall for large datasets. For the three disease topic/point-of-view pairings whose initial citation input exceeded 1000 (the drug treatment topics of arterial hypertension, diabetes mellitus type 2, and congestive heart failure) average recall was 0.916.

### Drug treatment outputs

Combo algorithm-enhanced dynamic summarization outperformed conventional summarization and the baseline method in recall, but was outperformed by conventional summarization in terms of precision. Combo summarization achieved 0.85 average recall, and 0.38 average precision. The conventional schema produced average recall and precision scores of 0.59 and 0.71. Both dynamic summarization and conventional summarization outperformed the baseline method, which produced average recall and precision scores of 0.23 and 0.31. Based on these findings, if a clinician wished to locate the maximum amount of drug treatment options using one of these three methods, Combo would be the better choice. On the other hand, the new method is less precise, but this effect is moderated by the visualization tool that Semantic MEDLINE offers. Visualization conveniently presents all citation data (including the text of the abstract itself) that are relevant to an Intervention X_TREATS_disease Y relationship in an easily viewed, reader- friendly display. Viewed in context, clinicians can quickly discard irrelevant treatments. We would argue that recall is more critical in clinical browsing than precision. The cognitive load required to dismiss a false positive is lower than trying to deduce a missing (false negative) treatment. We chose to use the standard F_1_-score because it is more conventional, but if we weight recall more, in line with the argument above, then the Combo summarization would be quite competitive with the conventional technique.

### Prevention outputs

Combo summarization was less effective in identifying preventive interventions in the relevant reference standards, producing an average recall of 0.66 and an average precision rate of 0.33. There are two obvious possibilities for this diminished efficiency. First, the citation sets were substantially smaller than three of the four drug treatment citation sets, thus providing less initial data. As with most statistical techniques, larger sample sizes tend to lead to better performance. Second, preventive interventions described in text are often more general than drug therapies. For example, “lifestyle changes” may be more difficult to interpret in the SemRep phase. Also, the lower inter-annotator agreement scores suggest that clinicians are less apt to agree on prevention standards. This may also be reflected in the professional literature. Dynamic summarization with the Combo algorithm outperformed the baseline methodology, which produced an average recall of 0.27 and an average precision of 0.25. This suggests that dynamic summarization is a superior alternative to directly reviewing PubMed citations for identifying preventive interventions.

### Error analysis

We classified false positive findings by type, and false negative findings by the first sequential data source (i.e., PubMed, SemRep output, dynamic summarization output) that did not include them.

#### *False positives*

Most of the false positives for both drug treatment and prevention points-of-view could be classified as unproductive general subject arguments; pharmaceuticals or supplements not included in the relevant reference standards; or other therapies not included in the relevant reference standards. In the prevention data, pharmaceuticals or supplements not included in the relevant reference standards accounted for 62.5% of all false positives, while unproductive general subject arguments and other therapies not included in the relevant reference standards accounted for 17.5% and 15.5%, respectively. In the drug treatment data, pharmaceuticals or supplements not included in the relevant reference standard accounted for an even greater percentage of false positives at 73.7%, while unproductive general subject arguments and other therapies not included in the relevant reference standard accounted for 14.2% and 12%. There are several possible reasons why there was such a high percentage of non-reference standard pharmaceutical or supplement false positives. Initial citation retrieval was not limited by a beginning publication date. In other words, all search queries retrieved relevant citations for as far back in time as PubMed made available. Therefore, information retrieval likely included older drugs which had been replaced by newer medications as preferred treatments. Also, we used a single data source in creating the reference standard. If we had included recommendations from other decision support tools in addition to those from DynaMed, the final reference standard might have included other treatments found within this false positive classification. Another data trend substantially contributed to reduced precision. Subject arguments that occurred two times or less in an output for a given disease topic/point-of-view pairing accounted for 69.7% of all false positives. If these arguments were removed from the output, average precision for both drug treatment and preventive intervention data combine would increase from 35% to 80%, with a proportionately small effect on recall.

#### *False negatives*

Because Semantic MEDLINE is a pipeline application, data loss can be tracked by documenting the first sequential process (among PubMed retrieval, SemRep, and dynamic summarization) that does not include a reference standard intervention. We applied this method in analyzing false negative interventions to determine which process “lost” the desired data. In tracking the 23 false negatives that addressed a drug treatment point-of-view, PubMed retrieval did not garner 43.5% (10 false negatives); SemRep output did not include 47.8% (11 false negatives); and dynamic summarization did not identify 8.7% (2 false negatives). False negatives emerging from the prevention point-of-view data were slighted more balanced. In this case, PubMed retrieval did not include 41.2% (7 false negatives) while SemRep output did not include 35.3% (6 false negatives) and dynamic summarization output did not include 23.5% (4 false negatives). However, in analyses for both points-of-view, dynamic summarization performed better than the other two processes. Visualization output was not included; it was considered irrelevant, since it automatically includes all output from summarization.

### PubMed retrieval volume and performance

Performance measurements suggest a system preference for larger citation input. Among search queries pairing the disease topics with the drug therapy subheading, the only query resulting in a relatively small amount of citations (the pneumonia pneumococcal query) also lead to comparatively diminished performance. System performance for pneumococcal pneumonia drug treatment data produced only 0.65 recall, while the other disease topic/drug treatment pairings achieved 0.89 or higher recall. System performance for prevention had similar results, with recall ranging from 0.50 to 0.76, with overall fewer citations than the drug treatment data. However, in a pilot project the system produced 100% recall for prevention data on a single disease topic (acute pancreatitis), with only 156 citations [41]. We conclude that citation volume can be a factor for some clinical topics, but not for all of them. In cases like acute pancreatitis, where therapeutic options are narrow, the system can perform comparably despite a relatively sparse citation set.

### Reference standards and system performance

We selected DynaMed as the source for our reference standards because it ranked among the top three point-of-care information delivery products in a recent study by Banzi and colleagues [37]. We chose DynaMed instead of one of the other top-ranking products, EBM Guidelines [42] and UpToDate [43], because we did not have access to EBM Guidelines, and DynaMed’s presentation format was superior to that of UpToDate for the purposes of this study. However, DynaMed is not necessarily an all-inclusive source of effective interventions. By Banzi’s own disclosure, no decision support product proved to be “the best”, at least according to his criteria. Reference standards including recommendations from all three products may be more comprehensive, and shed better light on all three summarization methodologies’ recall and precision performance.

### Comparisons to other methods

It is difficult to perform a one-to-one comparison with other text summarization methods, due to the unique reference standards we used to evaluate dynamic summarization. However a performance comparison with other applications that implement a conventional point-of-view refinement may offer valuable insight. Zhang and her colleagues incorporated an application utilizing degree centrality into Semantic MEDLINE with conventional treatment summarization [31]. The degree centrality component was applied after summarization. This approach achieved 73% precision and 72% recall when evaluated with a handcrafted reference standard of answers to disease properties. Fiszman and colleagues created an application for identifying citations valuable to clinical guideline creation [44]. Using guideline-oriented questions, they created a set of rules that functioned similarly to conventional summarization, to achieve a type of point of-view filtering for guideline-relevant data. This application achieved 40% recall and 88% precision using another manually-assembled reference standard of relevant and non-relevant citations. Combo-enhanced dynamic summarization achieved lower precision than these methods. However, its combine average recall for both drug treatment and preventive interventions exceeds that of both degree centrality and clinical guideline citation identification. In future work, when the precision-improving adjustments are applied, precision may exceed these products.

### Limitations

There are limitations in this study. It explores summarization for only two points-of-view (prevention and drug treatment) for the single task of decision support. However, an earlier study examined Combo-enhanced dynamic summarization for a genetic disease etiology point-of-view, within the task of secondary genetic database curation [19]. The curation study revealed improved summarization performance for that task. In this current study, we examined dynamic summarization for just four disease topics. However, a pilot project [41] featuring three different disease topics (acute pancreatitis, coronary artery disease, and malaria), again within the context of preventive intervention decision support, produced slightly superior results. This creates optimism that this text summarization method may enable others to locate decision support data. The initial search queries that retrieved the PubMed citations utilized controlled vocabulary terms. Keyword queries may offer additional insight to the dynamic Semantic MEDLINE application. Finally, we evaluated system output with recommendations garnered from a single commercial decision support product. Comparing performance to other decision support sources may shed further light on Combo-enhanced dynamic summarization as a potential decision support tool.

## Conclusion

In order to evaluate the performance of a new dynamic text summarization extension (Combo) within Semantic MEDLINE, we applied it, plus conventional Semantic MEDLINE, and a baseline summarization methodology (designed to mimic manual clinical review) to a clinical decision support task. We chose four disease topics and processed PubMed citations addressing their drug treatment and prevention. We processed the citations with SemRep, an application that transforms PubMed text into semantic predications. We then processed the SemRep output using the three summarization methodologies.

An evaluation using reference standards (clinically vetted DynaMed) showed that the new summarization method outperformed the conventional application and baseline methodology in terms of recall, while the conventional application produced the highest precision. Dynamic and conventional summarization were superior to the baseline methodology. These findings imply that the new text summarization application holds potential in assisting clinicians in locating decision support information.

## Abbreviations

NLP: Natural language processing; UMLS: Unified medical language system.

## Competing interests

The authors declare that they have no competing interests.

## Authors’ contributions

TEW designed the study; downloaded the citations; oversaw data processing with SemRep, the conventional treatment summarization schema, and the dynamic summarization application; built the baseline measurements; coordinated reference standard construction; performed the manual data evaluation; and wrote the original manuscript. MF guided the data evaluation and provided essential manuscript revisions. JFH contributed to the Combo algorithm design by suggesting use of the RlogF metric; provided guidance in the reference standard vetting process; and also provided essential manuscript revisions. All authors read and approved the final manuscript.

## Pre-publication history

The pre-publication history for this paper can be accessed here:

http://www.biomedcentral.com/1472-6947/12/41/prepub
